# Characterizing the Contributions of Various Clostridium perfringens Enterotoxin Properties to *In Vivo* and *In Vitro* Permeability Effects

**DOI:** 10.1128/msphere.00276-22

**Published:** 2022-09-07

**Authors:** Archana Shrestha, Mauricio A. Navarro, Juliann Beingesser, Anibal G. Armien, Francisco A. Uzal, Bruce A. McClane

**Affiliations:** a Department of Microbiology and Molecular Genetics, University of Pittsburgh School of Medicine, Pittsburgh, Pennsylvania, USA; b California Animal Health and Food Safety Laboratory System, School of Veterinary Medicine, University of California Davis, San Bernardino, California, USA; c Instituto de Patologia Animal, Facultad de Ciencias Veterinarias, Universidad Austral de Chile, Valdivia, Chile; University of Michigan-Ann Arbor

**Keywords:** *Clostridium perfringens*, enterotoxin, enterotoxemia, permeability alterations, pore formation, Caco-2 cells

## Abstract

Clostridium perfringens enterotoxin (CPE) is thought to cause lethal enterotoxemia when absorbed from the intestinal lumen into the circulation. CPE action sequentially involves receptor-binding, oligomerization into a prepore, and pore formation. To explore the mechanistic basis by which CPE alters permeability, this study tested the permeability effects of several recombinant CPE (rCPE) species: rCPE and rCPE_C186A_ (which form pores), rC-CPE and rCPE_D48A_ (which bind to receptors but cannot oligomerize), rCPE_C186A/F91C_ (which binds and oligomerizes without pore formation), and rCPE_Y306A/L315A_ (which has poor receptor-binding ability). On Caco-2 cells, i) only rCPE and rCPE_C186A_ were cytotoxic; ii) rCPE and rCPE_C186A_ affected transepithelial resistance (TEER) and 4 kDa fluorescent dextran (FD4) transit more quickly than binding-capable, but noncytotoxic, rCPE variants; whereas iii) rCPE_Y306A/L315A_ did not affect TEER or FD4 transit. Using mouse intestinal loops, rCPE (but not noncytotoxic rC-CPE, rCPE_D48A_ or rCPE_Y306A/L315A_) was lethal and caused intestinal histologic damage within 4 h. After 2 h of treatment, rCPE was more strongly absorbed into the serum than those noncytotoxic rCPE species but by 4 h rC-CPE and rCPE_D48A_ became absorbed similarly as rCPE, while rCPE_Y306A/L315A_ absorption remained low. This increased rC-CPE and rCPE_D48A_ absorption from 2 to 4 h did not involve a general intestinal permeability increase because Evans Blue absorption from the intestines did not increase between 2 and 4 h of treatment with rC-CPE or rCPE_D48A_. Collectively, these results indicate that CPE receptor binding is sufficient to slowly affect permeability, but CPE-induced cytotoxicity is necessary for rapid permeability changes and lethality.

**IMPORTANCE** Clostridium perfringens enterotoxin (CPE) causes lethal enterotoxemia when absorbed from the intestines into the bloodstream. Testing recombinant CPE (rCPE) or rCPE variants impaired for various specific steps in CPE action showed that full CPE-induced cytotoxicity causes rapid Caco-2 monolayer permeability alterations, as well as enterotoxemic lethality and rapid CPE absorption in mouse small intestinal loops. However, receptor binding-capable, but noncytotoxic, rCPE variants did cause slow-developing *in vitro* and *in vivo* permeability effects. Absorption of binding-capable, noncytotoxic rCPE variants from the intestines did not correlate with general intestinal permeability alterations, suggesting that CPE binding can induce its own uptake. These findings highlight the importance of binding and, especially, cytotoxicity for CPE absorption during enterotoxemia and may assist development of permeability-altering rCPE variants for translational purposes.

## INTRODUCTION

The action of Clostridium perfringens enterotoxin (CPE) commences with its binding to claudin receptors (1–3). This binding creates a small (~90 kDa) complex containing CPE, a receptor claudin, and a nonreceptor claudin (4). Six small CPE complexes then oligomerize to form an ~500 kDa prepore on the host cell membrane surface (4). Each CPE molecule resident in the prepore then extends a beta hairpin loop to create a beta barrel pore in the plasma membrane (5, 6). Low CPE doses form only small numbers of CPE pores, resulting in a limited calcium influx and modest calpain activation that leads to cell death via classical caspase-3 mediated apoptosis (7, 8). Treatment with higher CPE concentrations causes formation of many CPE pores, more calcium influx and stronger calpain activation that induces cell death by necroptosis (7–9). In rabbit small intestinal loops, CPE cytotoxicity results in luminal fluid accumulation and histologic damage, including villus shortening and epithelial desquamation (10).

CPE is a 35 kDa single polypeptide consisting of two domains (11, 12), both essential for cytotoxicity. The C-terminal domain binds to claudin receptors (13–15). The N-terminal domain mediates two functions required for pore formation, i) the region surrounding residue 48 is involved in oligomerization to form the CPE prepore (6, 16, 17), whereas ii) the region extending from amino acid residues 80 to 106 unwinds into a beta hairpin to make the CPE pore (5, 6).

CPE is an essential contributor to C. perfringens type F strain pathogenicity (18, 19). Type F strains cause 5–10% of antibiotic-associated diarrhea cases (20), as well as C. perfringens type F food poisoning, the 2nd most common bacterial foodborne disease in the United States (21). Although symptoms of this food poisoning are typically limited to diarrhea and abdominal cramps that self-resolve within 24 h, fatalities can occur in the elderly (21). More recently, it has become recognized that type F food poisoning can also be lethal in younger, physically healthy individuals suffering from medication-induced severe constipation or fecal impaction at the time of infection by a type F strain (22–24). Those preexisting conditions are thought to block the intestinal flushing effects of CPE-induced diarrhea, leading to a prolonged contact between CPE and the intestines (22) that likely increases disease severity. Supporting this view, CPE causes lethal enterotoxemia in mouse small intestinal loops, which mimic the static intestinal situation in people with fecal impaction or severe constipation (25). This mouse enterotoxemia involves absorption of CPE from the intestines into the circulation, where it then likely damages organs such as the liver. This damage results in lethal hyperpotassemia (25), which probably leads to cardiac arrest (26).

In addition to CPE’s role in pathogenesis, there is considerable interest in exploiting this toxin for translational applications (27–30). CPE or its derivatives have been explored for use in cancer therapy, imaging, or diagnosis (27). These proteins are also being explored for use as mucosal adjuvants and for drug delivery across a mucosal epithelium (28–33).

The ability of CPE to alter epithelial permeability is relevant for its absorption from the intestinal lumen during enterotoxemia and for translational applications but the mechanisms involved in CPE-induced permeability alterations have not been well-studied. CPE cytotoxicity, which results from pore formation (5), might be expected to impact the permeability of cultured epithelial cell monolayers or the intestines, including CPE absorption. However, cytotoxicity is not absolutely required for this toxin to affect the permeability of cultured epithelial cell monolayers (i.e., rC-CPE, a recombinant version of the noncytotoxic, binding-capable C-terminal domain of CPE) alters transepithelial electrical resistance (TEER) of, and fluorescent dextran 4 kDa (FD4) transit across, human enterocyte-like Caco-2 cell monolayers (34). rC-CPE also increases small molecule permeability in rat small intestine (34).

Many mechanistic questions remain regarding the permeability-altering properties of CPE or its derivatives. For example, which steps in CPE action are important for the toxin to efficiently alter permeability in highly confluent Caco-2 cell monolayers or the small intestine? Or which CPE properties are needed to cause lethal enterotoxemia? To dissect the contribution of various CPE properties to this toxin’s *in vitro* permeability-altering effects, including its own intestinal absorption *in vivo*, the current study utilized rCPE variants blocked at different steps in CPE action. Each variant was compared to fully cytotoxic rCPE for its ability to alter permeability in highly confluent Caco-2 cell monolayers or the intestine and to induce their absorption from the intestinal lumen into the circulation of mice.

## RESULTS

### Characterization of rCPE_Y306A/L315A_ binding and oligomerization properties.

**FIG 1:**
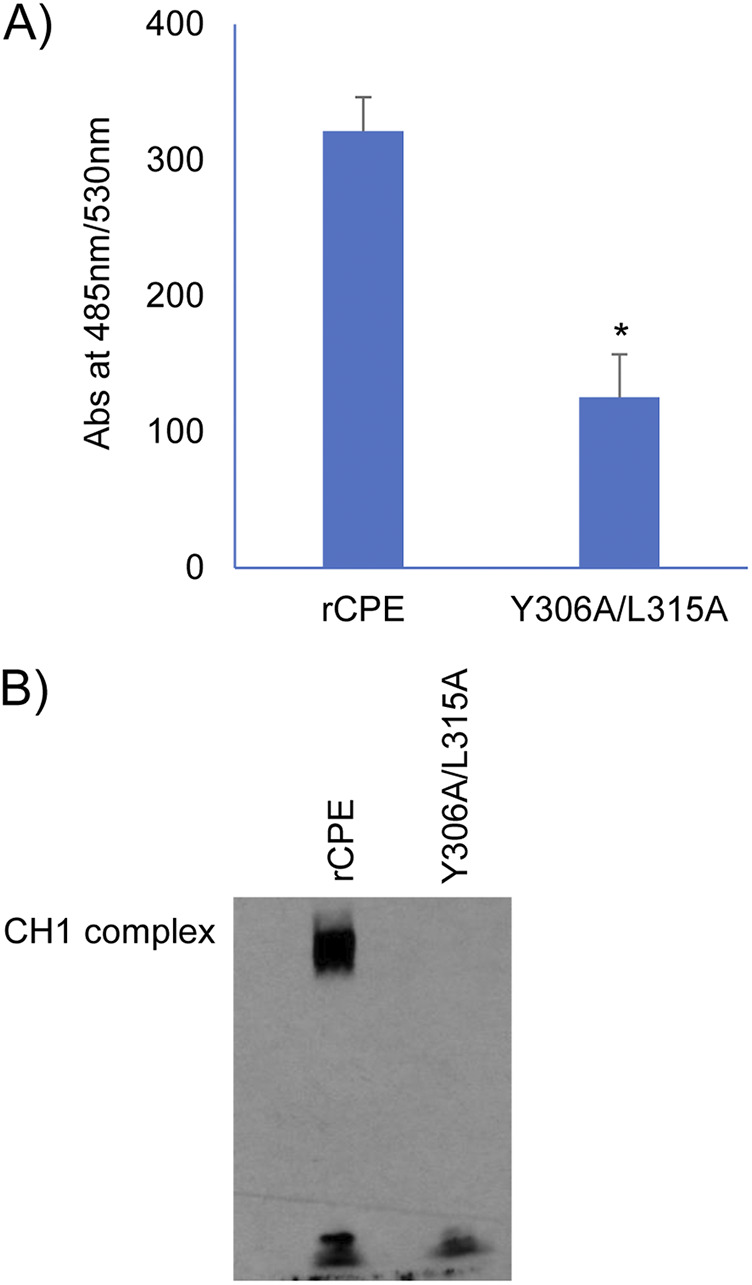
Characterization of rCPE_Y306A/L315A_ properties. (A) Comparison of the binding of AF488-labeled rCPE_Y306A/L315A_ versus AF488-labeled rCPE to Caco-2 cells. Highly confluent Transwell cultures of Caco-2 cells were treated for 1 h at 37°C with HBSS containing 5 μg/mL of either AF488-labeled rCPE species. The cells were then washed twice with HBSS buffer and lysed with RIPA buffer. Lysates were collected to measure fluorescence using a multiplate reader (BioTek, Winooski, VT) with excitation and emission of 485 nm. Results shown are the mean of three repetitions. Error bars show SD. * indicates statistical significance (*P* < 0.05). (B) Western blot analysis of CH-1 pore complex formation by rCPE_Y306A/L315A_. Highly confluent Transwell cultures of Caco-2 cells were treated for 1 h at 37°C with HBSS containing 5 μg/mL of either rCPE or rCPE_Y306A/L315A_. The cells were then washed, lysed and processed for CPE Western blotting. Note the presence of the CPE CH-1 pore complex only in cells treated with rCPE. The blot shown is representative of three repetitions.

The binding abilities (Table 1) of rCPE, rCPE_C186A_, rCPE_D48A_, rCPE_C186A/F91C_ and the C-terminal CPE domain (rC-CPE) were demonstrated previously (5, 13, 16, 17) in our laboratory. Others reported that rCPE_Y306A/L315A_ shows poor binding ability (35), so the current study first sought to confirm that prior conclusion by comparing the Caco-2 cell binding abilities of Alexa-Fluor 488 (AF-488)-labeled rCPE_Y306A/L315A_ vs AF-488-labeled rCPE, which is capable of binding to Caco-2 cells. This experiment (Fig. 1A) detected significantly reduced binding by AF-488-labled rCPE_Y306A/L315A_ compared to AF-488-labeled rCPE.

**TABLE 1:** rCPE species used in this study

rCPE species	Properties
Cytotoxic rCPE species	
rCPE	Fully cytotoxic toxin that binds, oligomerizes and forms pores (5, 6)
rCPE_C186A_	Fully cytotoxic rCPE variant that binds, oligomerizes and forms pores (5)
Noncytotoxic rCPE variants	
rC-CPE	C-terminal domain of rCPE; i.e., amino acids 184–319 of native CPE that binds but cannot oligomerize (43)
rCPE_D48A_	Binding-capable but cannot oligomerize (6)
rCPE_C186A/F91C_	Derived from rCPE_C186A_; binds and oligomerizes but does not form pores (5)
rCPE_Y306A/L315A_	Binds poorly (35) and this study

Because rCPE_Y306A/L315A_ still showed some, although significantly reduced, binding to Caco-2 cell monolayers, an experiment was performed to assess the ability of this variant to form the CH-1 CPE pore complex required for CPE-induced cytotoxicity (5). As shown in Fig. 1B, treatment with rCPE_Y306A/L315A_ failed to form detectable levels of this CPE pore complex, while formation of this complex by rCPE treatment was readily detectable. These results are fully consistent with previous reports (35) that rCPE_Y306A/L315A_ cannot bind well to claudin receptors for the toxin, as required for CPE prepore and pore formation (16, 36).

### Cytotoxic properties of the rCPE species used in this study.

Several previous studies from our laboratory had characterized the cytotoxic properties of rCPE, rCPE_C186A_, rCPE_D48A_, rCPE_C186A/F91C_ and the CPE C-terminal binding domain [Table 1 and 5, 10, 13, 16]. However, the *in vitro* cytotoxic properties of rCPE_Y306A/L315A_ had not yet been tested in our assays, nor had the cytotoxic properties of all these rCPE species been directly compared head-to-head in the same study. Therefore, before assessing their permeability-altering effects, the current study first compared the cytotoxic properties of the Table 1 rCPE species on highly confluent monolayers of Caco-2 cells.

**FIG 2:**
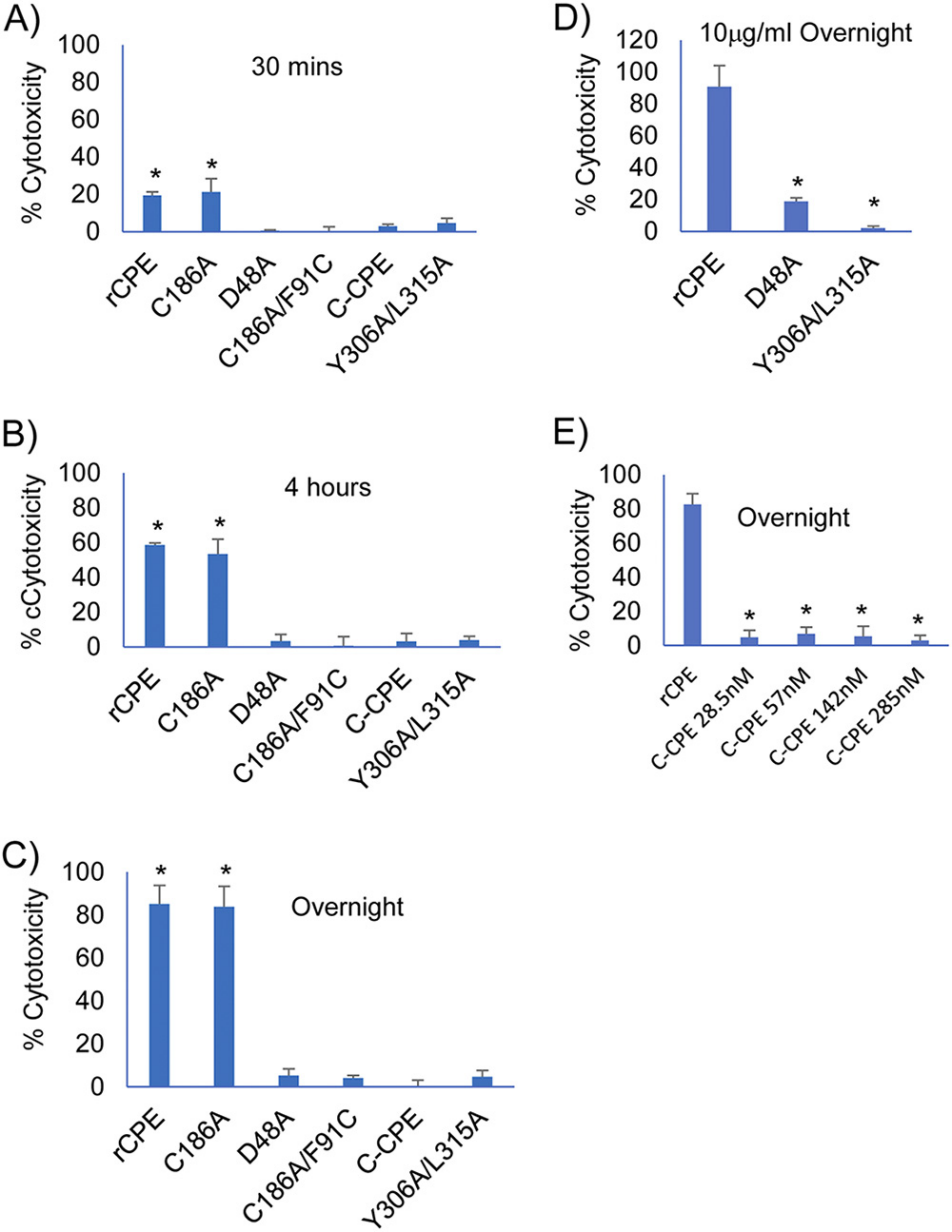
Cytotoxic properties of rCPE species. The cytotoxic effects of rCPE, rCPE_D48A_, rCPE_C186A/F91C_, rCPE_C186A_, rC-CPE, or rCPE_Y306A/L315A_ on highly confluent Transwell cultures of Caco-2 were measured after apical surface treatment for 0.5 h (A), 4 h (B), or overnight (C) (~24 h) at 37°C with HBSS containing a 28.5 nM concentration of each rCPE species. Also shown are the overnight cytotoxic effects of treatment with (D) HBSS containing either a 285 nM concentration of rCPE_D48A_ or rCPE_Y306A/L315A_ or (E) HBSS containing up to a 285 nM concentration of rC-CPE. After completion of these treatments, supernatants from each apical Transwell chamber were collected and cytotoxicity deduced using a LDH cytotoxicity assay (Roche). Result shown are the mean of three independent experiments. Error bars depict the SD. In panels A–C, * represents *P* < 0.05 compared to rCPE_Y306A/L315A_. In panels D and E, * represents *P* < 0.05 compared to rCPE.

Consistent with previous reports (5), treatment of highly confluent Caco-2 cell monolayers with a 28.5 nM (1 μg/mL) concentration of rCPE or rCPE_C186A_ caused rapid (i.e., within 30 min) development of significant cytotoxicity (Fig. 2A). The extent of that cytotoxicity then increased progressively thereafter (Fig. 2B and C). In contrast, none of the other rCPE species were cytotoxic at this same concentration, even when this treatment was extended overnight (Fig. 2C).

**FIG 3:**
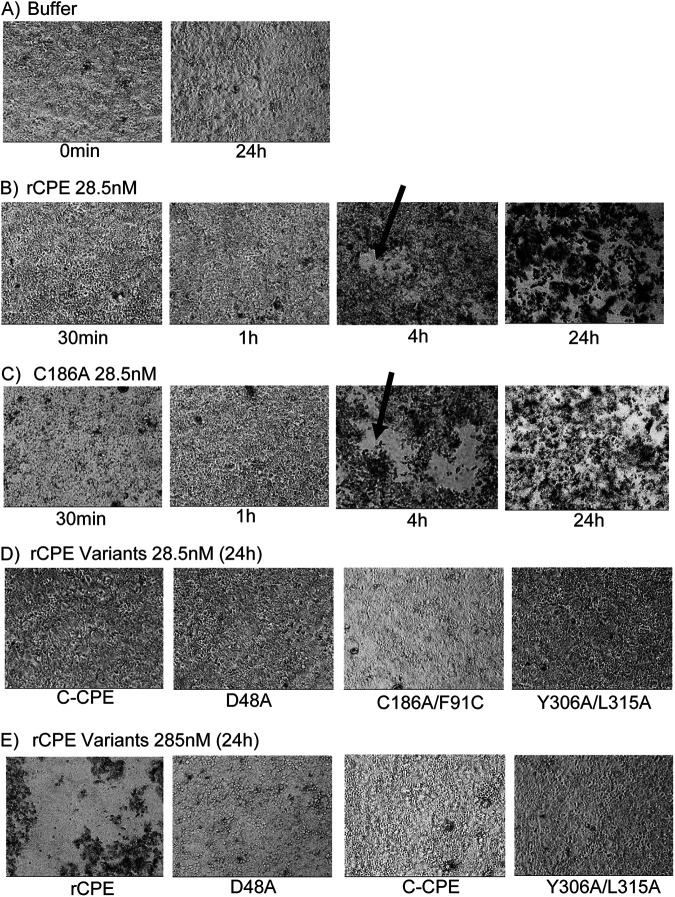
Photomicroscopy comparison of Caco-2 cell monolayer damage caused by rCPE species. The development of damage in highly confluent Caco-2 cell monolayers was visually monitored by microscopy for every experiment shown in Fig. 2. Those effects were also documented for representative wells by photomicroscopy, as shown in Fig. 3. Panel A shows a highly confluent monolayer at the beginning of the experiment and the absence of damage to that same monolayer after a 24 h of incubation in HBSS. Panel B shows the development of damage to a representative monolayer over a 24 h treatment period with HBSS containing 28.5 nM rCPE. Note the appearance of monolayer gaps between 1 and 4 h of treatment (black arrows) and complete monolayer destruction by 24 h which resulted in many floating, detached cells. Panel C demonstrates similar development of damage to a representative monolayer over a 24 h treatment period with HBSS containing 28.5 nM rCPE_C186A_ (black arrow indicates monolayer gaps after 4 h treatment), with many floating, detached cells present after 24 h of treatment. Panel D shows the absence of monolayer damage in representative wells after 24 h treatment with a 28.5 nM concentration of rC-CPE, rCPE_D48A_, rCPE_C186A/F91C_ or rCPE _Y306A/L315A_. Panel E demonstrates that overnight treatment with HBSS containing even high (285 nM) concentrations of rC-CPE, rCPE_D48A_ or rCPE _Y306A/L315A_ failed to cause monolayer damage, unlike the monolayer destruction caused by 24 h treatment with HBSS containing this same high concentration of rCPE, which included many floating, detached cells at this treatment time.

Damage to Caco-2 cell monolayers was also visually monitored by microscopy for all Fig. 2 experiments. Monolayers treated with Hank’s Balanced Salts Solution (HBSS, Mediatech) alone did not show any detectable damage after overnight (~24 h) treatment. However, treatment with HBSS containing a 28.5 nM concentration of rCPE or rCPE_C186A_ began causing monolayer damage, including cell rounding and cell detachment to form monolayer gaps, between 1 to 4 h of treatment. This damage became more severe after overnight treatment such that the monolayer was destroyed and nearly all cells had detached by that time point. In contrast, even overnight treatment with a 28.5 nM concentration of rCPE_D48A_, rCPE_C186A/F91C_ or rC-CPE did not produce detectable monolayer damage. Representative photomicrographs supporting these conclusions are shown in Fig. 3A–D.

Three rCPE variants, i.e., rC-CPE, rCPE_D48A_ and rCPE_Y306A/L315A_, that tested noncytotoxic in Fig. 2A experiments were obtainable in large amounts, allowing further testing of their cytotoxic effects under even more stringent treatment conditions, i.e., overnight treatment of confluent Caco-2 cell monolayers with up to a 10-fold higher (285 nM) concentration of that rCPE variant. However, even with these much harsher treatment conditions, no significant cytotoxicity was observed for rCPE_Y306A/L315A_ (Fig. 2D) or rC-CPE (Fig. 2E) and only limited (~12%) cytotoxicity was detected for rCPE_D48A_ (Fig. 2D). Damage to these Caco-2 cell monolayers was also monitored by microscopy. Monolayers treated overnight with HBSS containing a 285 nM concentration of rCPE showed extensive monolayer damage, including cell rounding and detachment of nearly all cells. In contrast, even overnight treatment with a 285 nM concentration of rCPE_D48A_, rCPE_C186A/F91C_ or rC-CPE did not produce detectable monolayer damage. Representative photomicrographs supporting these conclusions are shown in Fig. 3E.

### Effects of rCPE species on permeability properties of confluent Caco-2 cell monolayers.

**FIG 4:**
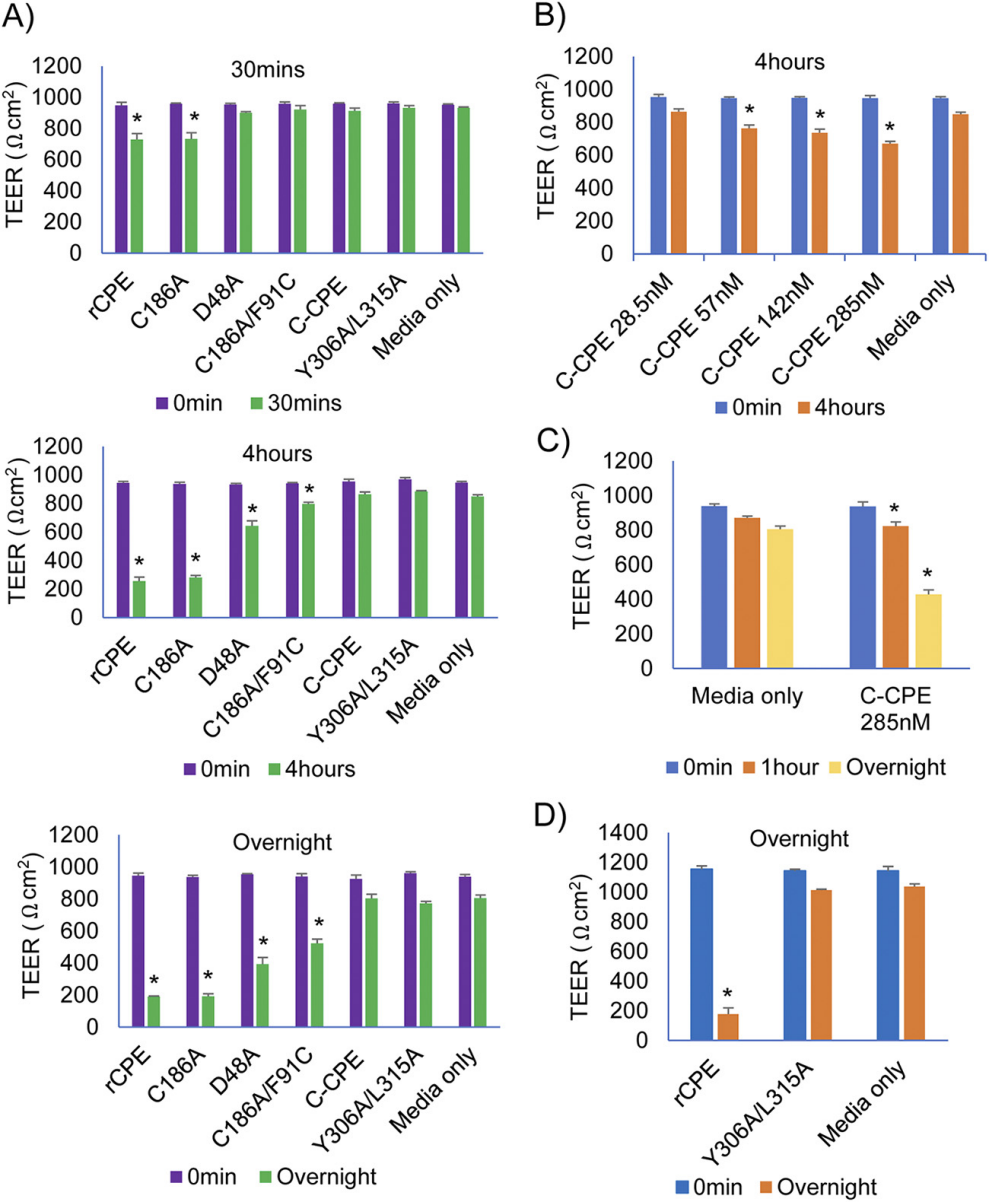
Effects of rCPE, rCPE_D48A_, rCPE_C186A/F91C_, rCPE_C186A_, rC-CPE or rCPE_Y306A/L315A_ on transepithelial electrical resistance (TEER) properties of Caco-2 cells. (A) Highly confluent Caco-2 cell monolayers grown in Transwells were treated on their apical (upper well) surface for 1 h at 37°C with medium (no serum) containing a 28.5 nM concentration of one rCPE species, as indicated. Transepithelial Electrical Resistance (TEER) was then measured at specified time points (overnight was ~24 h). (B) Concentration-dependent effects of rC-CPE on TEER in highly confluent Caco-2 cell monolayers grown in Transwells. Cultures were treated for 0 or 4 h with medium (no serum) containing the indicated concentration of rC-CPE and TEER was then measured. (C) Effects of longer treatment time with medium (no serum) containing a high concentration (285 nM) of rC-CPE on TEER in highly confluent Caco-2 cell monolayers grown in Transwells. (D) TEER effects of medium (no serum) containing a high (285 nM) dose treatment of rCPE_Y306A/L315A_ versus rCPE applied overnight to highly confluent Caco-2 cell monolayers grown in Transwells. Results shown in all panels are the average of three independent experiments. Error bars depict the SD. * represents *P* < 0.05 compared to control cell culture media.

To begin comparing their ability to alter the permeability of epithelial cell monolayers, the effects of various rCPE species on TEER were measured using highly confluent Caco-2 cell monolayers (Fig. 4). Treatment of these monolayers using a 28.5 nM concentration of either rCPE or rCPE_C186A_ began altering TEER within 30 min, with this effect then increasing throughout the experimental duration (Fig. 4A). In contrast, 30 min of treatment with a 28.5 nM concentration of any noncytotoxic rCPE variant did not significantly affect TEER (Fig. 4A). However, after 4 h of this treatment, rCPE_D48A_ or rCPE_C186A/F91C_ caused a significant decrease in TEER and those effects became stronger with overnight treatment (Fig. 4A). In contrast, even overnight treatment with a 28.5 nM concentration of rC-CPE or rCPE_Y306A/L315A_ did not significantly alter TEER in Caco-2 monolayers (Fig. 4A).

When the effects of higher concentrations of rC-CPE or rCPE_Y306A/L315A_ on TEER were similarly evaluated, 4 h of treatment using a 2-fold higher (57 nM) concentration of rC-CPE was sufficient to significantly decrease TEER (Fig. 4B). Furthermore, treatment using a 10-fold higher (285 nM) concentration of rC-CPE significantly dropped TEER after 1 h, while overnight treatment using this high rC-CPE dose caused an ~50% reduction in TEER (Fig. 4C). However, even overnight treatment using a 285 nM concentration of rCPE_Y306A/L315A_ did not significantly affect TEER (Fig. 4D).

**FIG 5:**
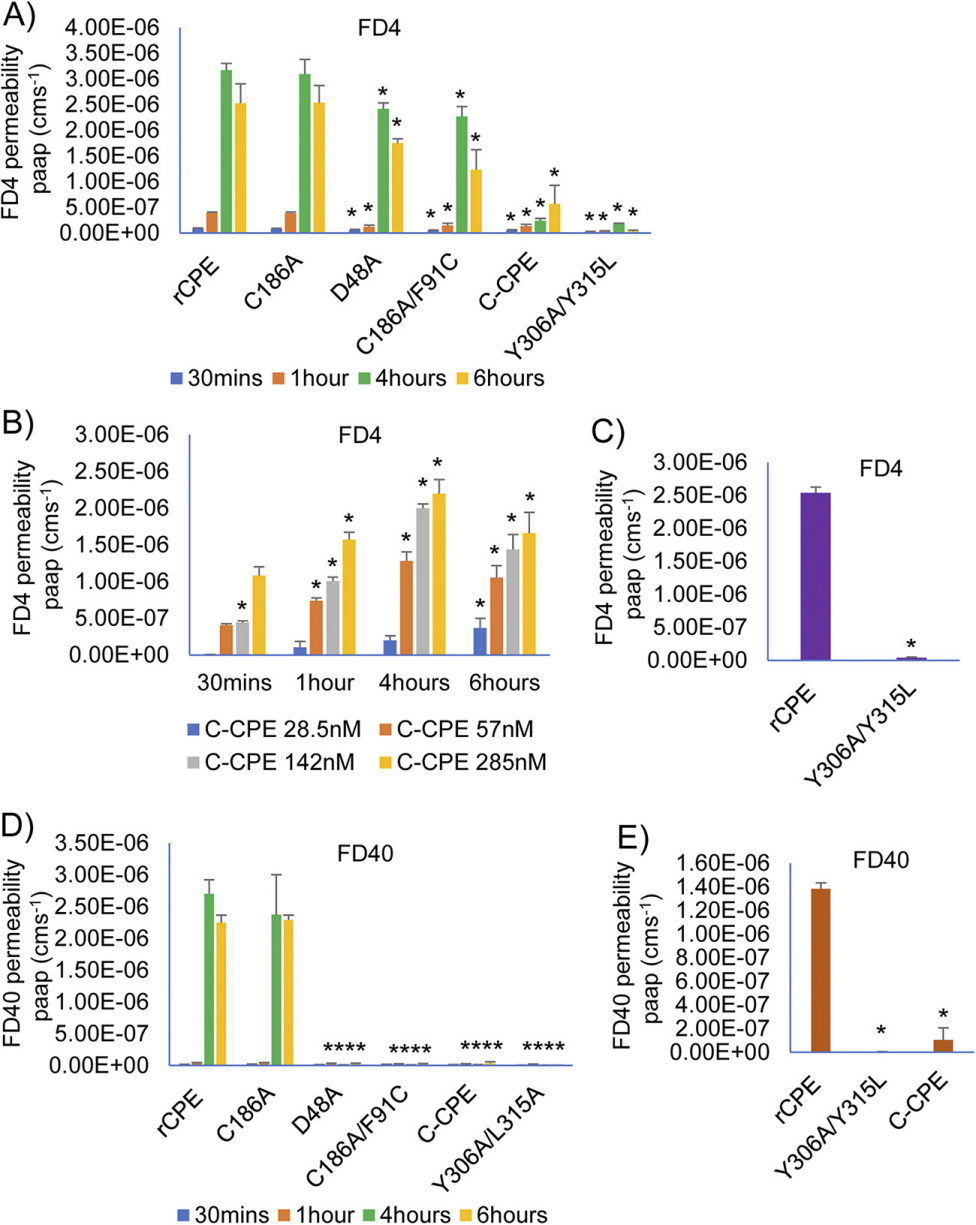
Effects of rCPE, rCPE_D48A_, rCPE_C186A/F91C_, rCPE_C186A_, rC-CPE or rCPE_Y306A/L315A_ on FD4 or FD40 transit across highly confluent Caco-2 cell monolayers. Two-week-old, highly confluent Caco-2 cell monolayers grown in 12 mm^2^ Transwell plates were treated, for specified times, on their apical (upper well) surface with HBSS containing one rCPE species plus either FITC-labeled 4KDa dextran (FD4, panels A–C) or FITC-labeled 40 kDa dextran (FD40, panels D and E) at a 1 mg/mL final concentration. FD4 transit effects shown include treatment with (A) 28.5 nM each species for 0.5–6 h, (B) specified concentrations of rC-CPE for 0.5–6 h, or (C) overnight (~24 h) treatment with 285 nM rCPE or rCPE_Y306A/L315A_. Panel D shows FD40 transit effects of 28.5 nM concentration of each rCPE species for 0.5–6 h of treatment, whereas (E) shows FD40 transit effects of overnight (~24 h) treatment with 285 nM concentration of rCPE, rCPE_Y306A/L315A_ or rC-CPE. After treatment, the amount of FD4 or FD40 present in the basal chamber was measured using a BioTek Synergy fluorescence multi-plate reader. Data are presented as the mean ±SD of three independent experiments. Error bars depict the SD. * in A, C, D, or E represents *P* < 0.05 versus rCPE. * in B represents *P* < 0.05 compared to buffer.

To further analyze whether these rCPE species affect epithelial cell monolayer permeability, experiments were performed to evaluate their effects on FD4 or fluorescent dextran 40 kDa (FD40) transit across highly confluent Caco-2 cell monolayers cultured in Transwells. After correction for background FD4 transit in Caco-2 cell monolayers incubated in buffer alone, the presence of a 28.5 nM concentration of rCPE or rCPE_C186A_ modestly increased FD4 transit within 0.5 h and this effect was statistically significant compared with treatment with noncytotoxic rCPE species (Fig. 5A). By 4 h of treatment, the presence of a 28.5 nM concentration of all tested rCPE species, except rC-CPE and rCPE_Y306A/L315A_, induced a substantial increase in FD4 transit, although this effect remained significantly stronger for rCPE and rCPE_C186A_ (Fig. 5A).

By contrast, even 6 h of treatment with a 28.5 nM concentration of rC-CPE or rCPE_Y306A/L315A_ caused little or no increase in FD4 transit (Fig. 5A). However, challenges with higher doses (>28.5 nM) of rC-CPE did increase FD4 transit, with those effects reaching statistical significance within 1 h, even using only a 2-fold higher rC-CPE concentration (Fig. 5B). In contrast, a 6-h treatment with a 285 nM concentration of rCPE_Y306A/L315A_ did not significantly increase FD4 transit (Fig. 5C).

Treatment using a 28.5 nM concentration of rCPE or CPE_C186A_ significantly affected FD40 transit within 4 h (Fig. 5D). However, none of the noncytotoxic rCPE variants altered FD40 transit within 6 h of challenge with a 28.5 nM concentration (Fig. 5D). Even 10-fold higher concentration of rC-CPE or rCPE_Y306A/L315A_ did not significantly affect FD40 transit by 6 h (Fig. 5E).

### Assessing the ability of rCPE species to cause lethal enterotoxemia.

The *in vitro* results presented above indicated that, for rCPE to affect Caco-2 cell monolayer permeability, i) receptor-binding is important, ii) CPE pore formation and cytotoxicity hastens the onset of permeability changes but is not required for those changes to occur, and iii) the N-terminal region of CPE contributes to permeability changes beyond its role in pore formation. To begin exploring whether similar CPE properties are also required for enterotoxemia and the intestinal absorption of this toxin, the lethal enterotoxemic properties of rCPE, rC-CPE, rCPE_D48A_, and rCPE_Y306A/315A_ were first compared.

**FIG 6:**
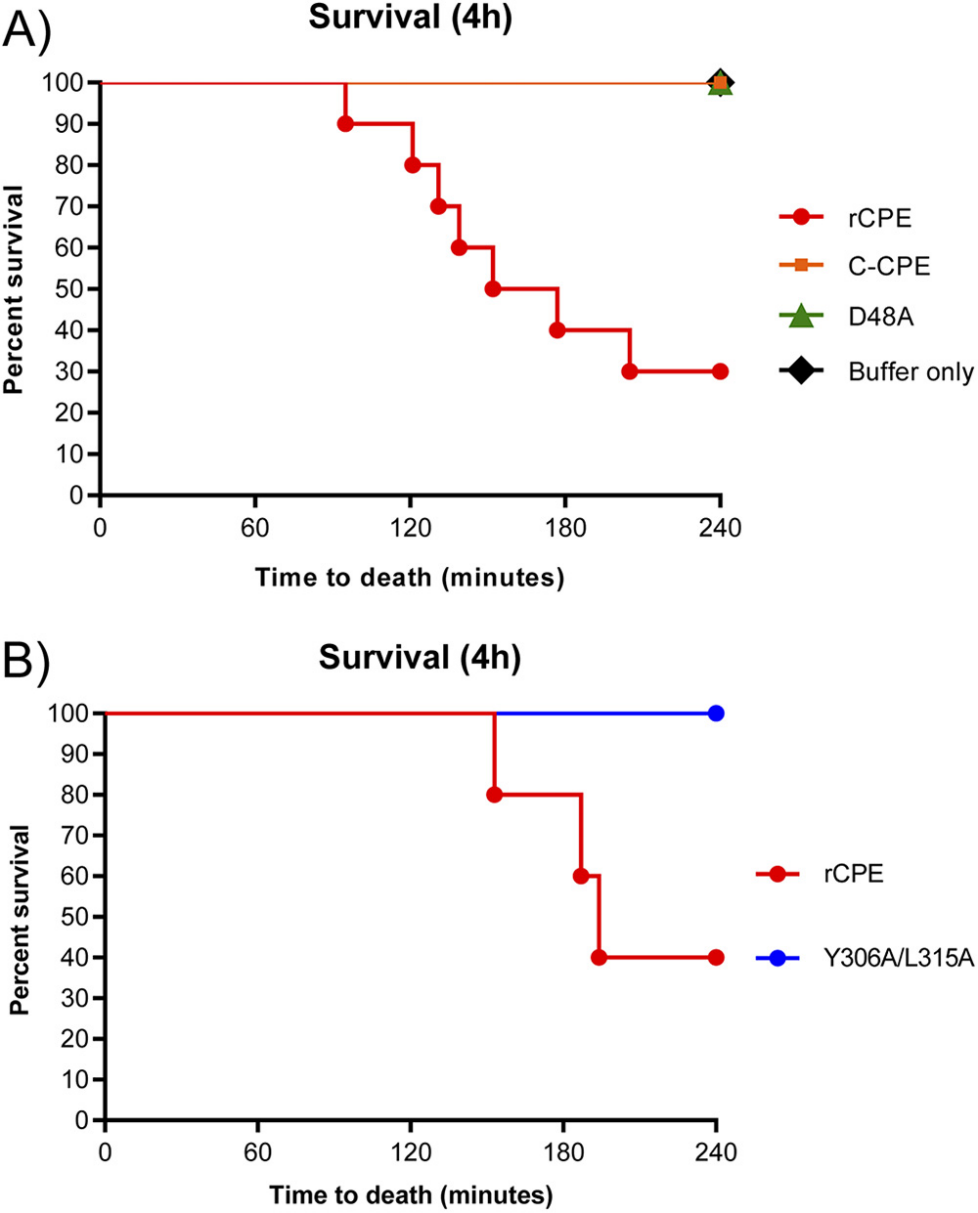
Comparison of lethal effects of rCPE, rCPE_D48A_, rC-CPE and rCPE_Y306A/L315A_ in mice. One mL of HBSS containing a 2.85 μM concentration of a specified rCPE species, or HBSS alone (buffer), was injected into a small intestinal loop constructed in a mouse. Lethality was observed up to 4 h postchallenge. Panel A shows results of an experiment using rCPE, rCPE_D48A_, rC-CPE or HBSS alone (*n* = 10 mice for each sample), whereas Panel B shows results of an experiment comparing the lethality of rCPE (*n* = 5 mice) versus rCPE_Y306A/L315A_ (*n* = 10 mice). rCPE was statistically different (*P* < 0.05) from all other samples in both panels.

In this experiment, mice were challenged via intra-small intestinal loop inoculation with HBSS or HBSS containing a 2.85 μM (equivalent to 100 μg/mL of CPE) concentration of one of those four rCPE species and then examined up to 4 h. Note that this concentration of rCPE species has pathophysiologic relevance, because diarrheic feces of CPE-associated food poisoning victims can contain <1 μg/mL to >100 μg/mL concentrations of this toxin (25, 37). Death was only observed in those mice whose intestinal loops had been challenged with HBSS containing rCPE (Fig. 6A and B). This lethal effect began ~2 h after initiation of the challenge and eventually 60–70% of mice treated with rCPE died (Fig. 6A and B).

### Absorption of rCPE species from the intestinal lumen into the circulation of mice.

**FIG 7:**
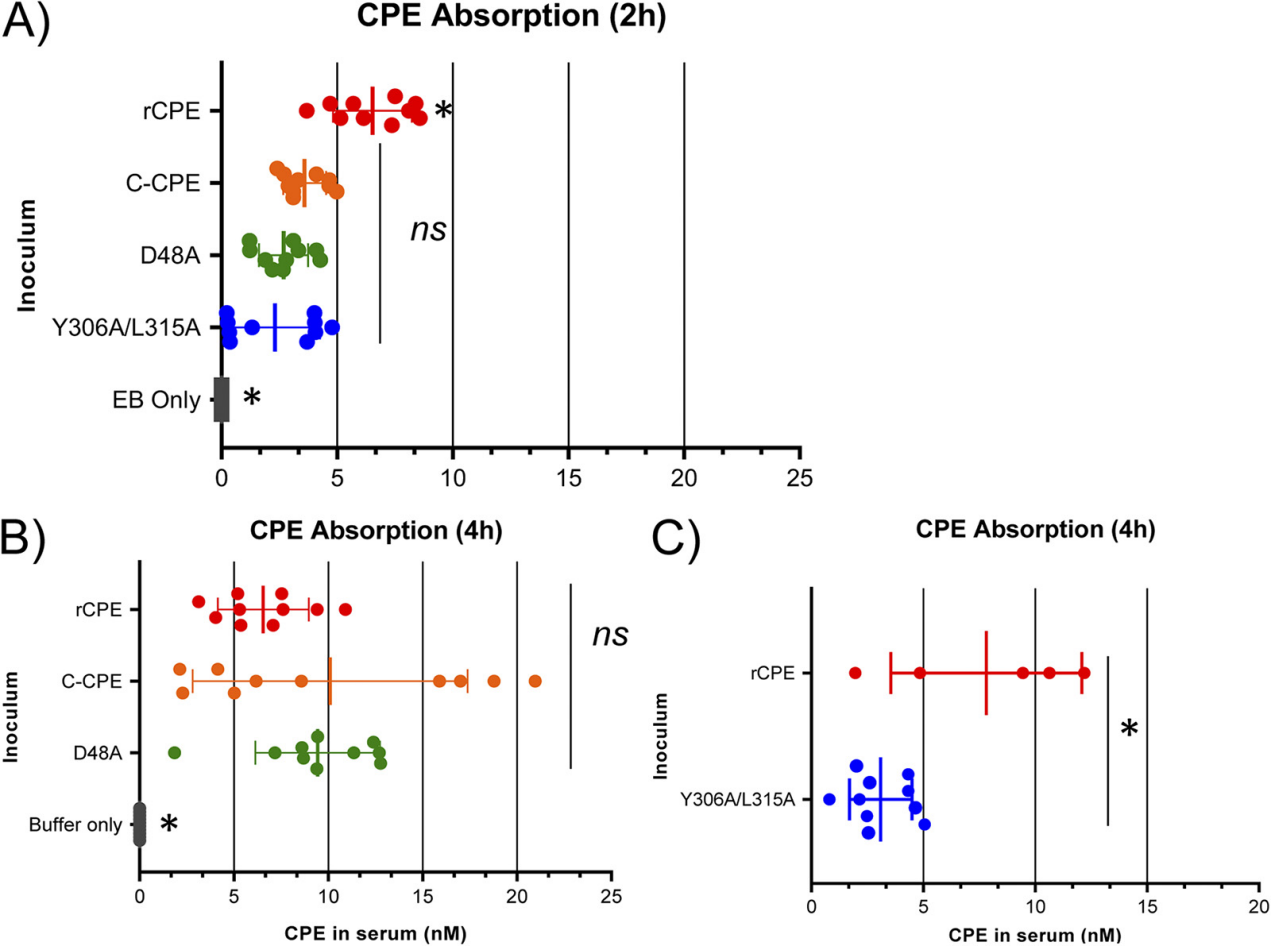
Absorption of rCPE species from the intestines into blood. One mL of HBSS (buffer; 4 h samples) or HBSS containing EB (EB only; 2 h samples) or those buffers plus a 2.85 μM concentration of each specified rCPE species was injected into a mouse small intestinal loop. After 2 h (A) or 4 h (B and C) blood was collected from the heart and rCPE species levels in serum were measured. * Indicates that these samples were significantly different from the other (ns) samples at *P* < 0.05. All results shown are for 10 mice per sample except for rCPE in Panel C, where 5 mice were used.

Experiments then directly addressed which CPE properties are necessary for absorption of this toxin from the intestinal lumen into the circulation, which is considered an important step for inducing the serum hyperpotassemia implicated in CPE-induced enterotoxemic lethality (25). Using sera collected from mice challenged with a 2.85 μM concentration of rCPE, rC-CPE, or rCPE_D48A_ (Fig. 7A), some absorption of those rCPE species was detected within 2 h. However, at this 2-h time point, rCPE was present at significantly higher serum levels compared to the noncytotoxic rCPE variants. Notably, when treatment times were extended to 4 h, the serum levels of the binding-capable, but noncytotoxic, variants rC-CPE or rCPE_D48A_ caught up to equal, if not exceed, the serum levels of rCPE (Fig. 7B). In contrast, when mice were challenged for 4 h with a 2.85 μM concentration of rCPE_Y306A/315A_, the serum levels of this poor-binding rCPE variant remained significantly less than for rCPE or the other tested variants and there was little change in rCPE_Y306A/315A_ serum levels between 2 and 4 h of treatment (Fig. 7C).

### Effects of rCPE species on intestinal histologic damage.

**FIG 8:**
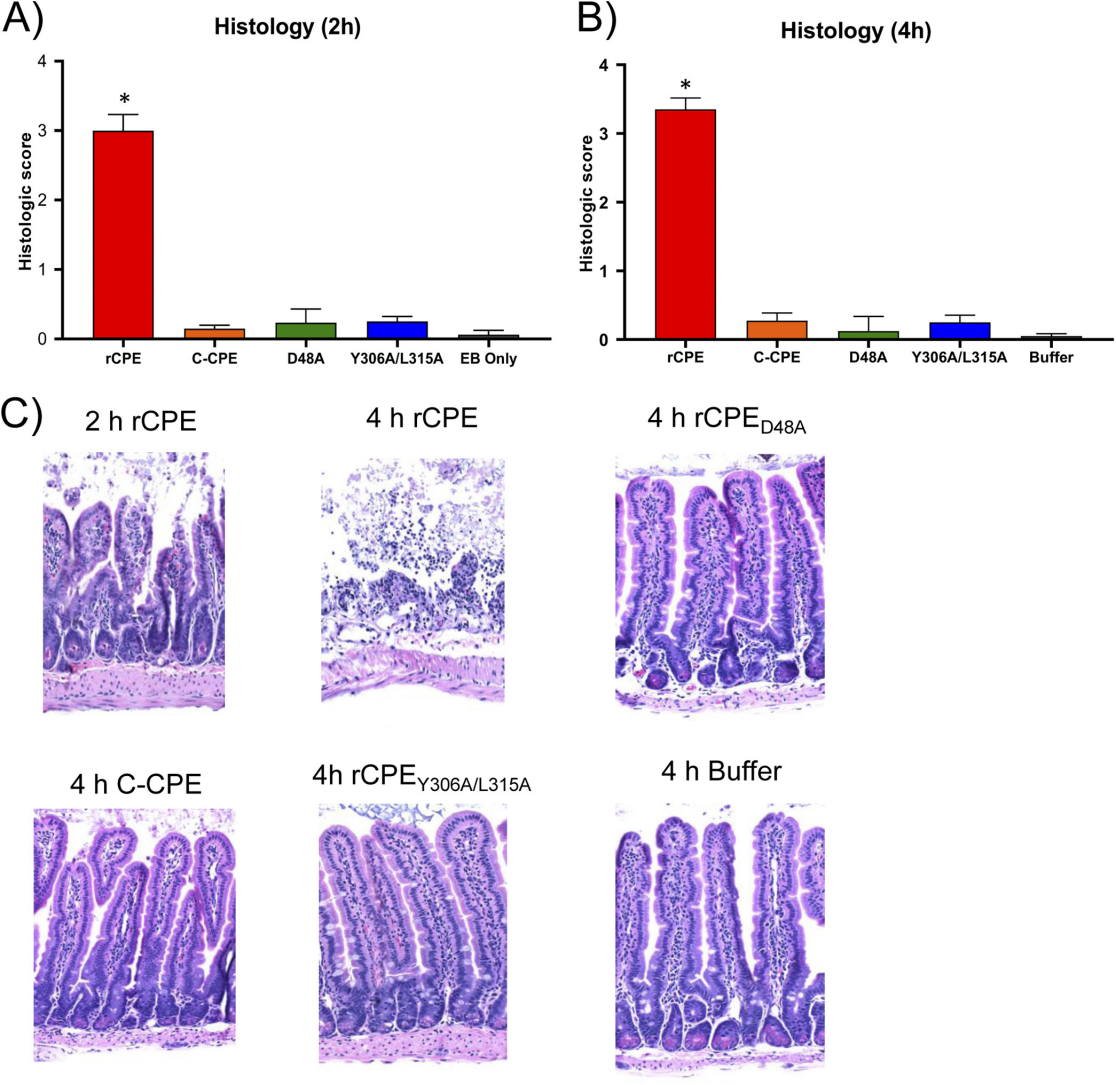
Comparison of intestinal histologic damage caused by rCPE species. Mouse small intestinal loops received an injection of 1 mL of HBSS containing EB (EB only; for 2 h samples) or HBSS alone (buffer; for 4 h samples) plus a 2.85 μM concentration of the specified rCPE species. After 2 or 4 h of treatment, or upon earlier spontaneous death, 4 μM-thick samples were sectioned and stained with hematoxylin and eosin (H&E). Panel A shows histologic scores (*n* = 10 mice) after 2 h of treatment, whereas Panel B scores these scores (*n* = 10 mice) after a 4-h treatment. Error bars indicate the standard error of the mean. Panel C shows representative photomicrograph results for each treatment condition. Note that rCPE produced intestinal damage at 4 h postchallenge but no histologic damage was visible even after 4 h treatment with other rCPE variants or buffer alone.

Fig. 7 results could be explainable if i) intestinal histologic damage is important for absorption of this toxin from the lumen and ii) relative to rCPE, the binding-capable, but noncytotoxic, rCPE variants cause more slowly developing intestinal histologic damage. To test those hypotheses, intestinal loops were treated with various rCPE species and examined histologically, as described in Materials and Methods. After either 2 or 4 h of treatment with a 2.85 μM concentration of rCPE, microscopic intestinal damage was apparent, with this damage increasing modestly, but significantly, between the 2 and 4 h time points (Fig. 8A and B). However, no significant microscopic damage was visible in intestinal loops after a 2 h (Fig. 8A and C) or 4 h (Fig. 8B and C) challenge with a 2.85 μM concentration of any tested noncytotoxic rCPE species (i.e., rC-CPE, rCPE_D48A_, or rCPE_Y306A/315A_). Similarly, no intestinal histologic damage was observed in loops treated with buffer or buffer containing only EB (Fig. 8A–C).

### General effects of rCPE species on intestinal permeability.

Another conceivable explanation for the Fig. 7 results could be that binding-capable, but noncytotoxic, rCPE variants eventually become well-absorbed because they induce a slow general breakdown in intestinal permeability that is independent of histologic damage. This possibility was tested by observing the effects of various rCPE species on Evans Blue (EB) absorption from the intestinal lumen, as an indicator of their effects on general intestinal permeability. After mouse small intestinal loops were treated for 2 or 4 h with HBSS containing 3% EB alone or 3% EB plus a 2.85 μM concentration of rCPE, C-CPE, rCPE_D48A_ or rCPE_Y306A/315A_, the amount of EB present in intestinal tissue was measured, as described in Materials and Methods.

**FIG 9:**
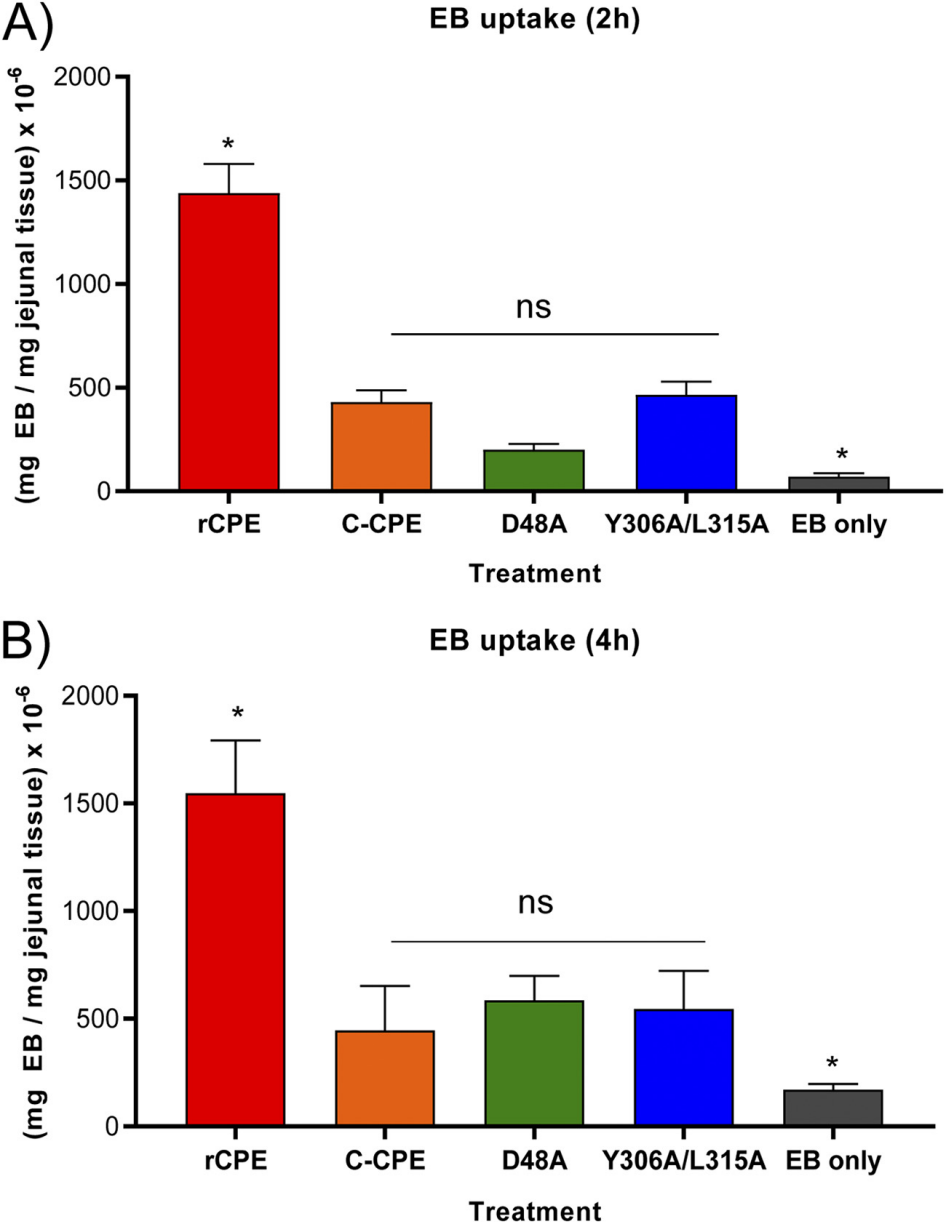
Effects of rCPE species on EB uptake. Mouse small intestinal loops received an injection of 1 mL of HBSS containing EB with or without a 2.85 μM concentration of the specified rCPE species. After 2 h (A) or 4 h (B) of treatment, EB uptake from the intestinal lumen into intestinal tissue was measured. * Indicates a statistically significant difference from the other samples, wheras ns indicates samples without a significant difference. Error bars show the standard error of the mean. Results show are for 10 mice for each challenge.

As shown in Fig. 9A, intestinal loops challenged for 2 h with rCPE showed significantly more EB uptake than loops similarly challenged with the other rCPE variants or loops receiving EB alone. At this 2 h time point, the noncytotoxic rCPE variants caused only a modest increase in EB uptake compared to treatment with buffer containing EB (no rCPE species). Notably, there was little change in EB uptake between 2 and 4 h (Fig. 9A and B) of treatment in animals challenged with any rCPE species, despite the sharp increase in intestinal absorption that occurs for the binding-capable, but noncytotoxic variants, between those two time points.

## DISCUSSION

Although the absorption of CPE from the intestines is thought to cause lethal enterotoxemia, the mechanistic basis of this process has been little studied. There is also interest in understanding how CPE alters epithelial permeability for translational applications, e.g., use of CPE or its derivatives for drug delivery (27–34). It was previously reported (38) that CPE can also affect the permeability of epithelial cell culture monolayers, including Caco-2 cell monolayers. Earlier studies had also determined that rC-CPE, which is not cytotoxic but binds to CPE receptors, can affect epithelial monolayer permeability *in vitro* and small intestinal permeability (34). However, the relative efficacy of rC-CPE vs rCPE permeability effects *in vitro* or *in vivo*, and the contribution of various steps in CPE action to those *in vivo* or *in vitro* permeability effects, had not yet been examined.

The current study has begun addressing these knowledge gaps by using rCPE and rCPE variants blocked at specific steps in CPE action. The first major conclusion provided by this approach is that receptor binding is an important contributor to most of the measured permeability effects, both *in vivo* and *in vitro*. Supporting this conclusion was the observation that the poor-binding rCPE_Y306A/315A_ variant was the only tested rCPE species that did not cause significant effects on TEER or FD4 transit in Caco-2 cell monolayers. By using rCPE_Y306A/315A_, it was also established that receptor binding enhances CPE intestinal absorption, i.e., by 4 h, significantly less of this rCPE variant was absorbed compared to the other rCPE species. These *in vivo* findings imply that, via receptor binding, CPE plays a major role in its own intestinal absorption, i.e., this uptake is not mainly a passive process involving unbound toxin. In only one experiment did receptor binding not closely correlate with increased permeability effects, i.e., rCPE_Y306A/315A_ and the noncytotoxic, but binding-capable, rCPE variants caused a similar, if small, increase in EB uptake above background. One possible explanation for this similarity could be that even the reduced binding activity of rCPE_Y306A/315A_ was sufficient to trigger this slight increase in EB uptake.

Using these rCPE and rCPE variants also allowed the current study to explore the contribution of various post-binding steps in CPE action to the *in vitro* permeability effects of this toxin. Results of those experiments confirmed previous reports (34) that cytotoxic activity is not essential for CPE to affect monolayer permeability of cultured epithelial cells (i.e., the binding-capable), but noncytotoxic rCPE variants eventually affected both TEER and FD4 transit in Caco-2 cell monolayers. However, the current study also established for the first time that cytotoxic activity significantly hastens the permeability effects of CPE on Caco-2 cell monolayers. Furthermore, although binding-capable, but noncytotoxic, rCPE variants increased small molecule (FD4) transit, only cytotoxic rCPE affected large molecule (FD40) transit in this *in vitro* model.

The current study also provided several additional new insights into CPE permeability effects on Caco-2 cell monolayers. First, even in the absence of pore formation and cytotoxicity, the N-terminal domain of the toxin was shown to contribute to CPE-induced permeability effects on Caco-2 monolayers. Specifically, the noncytotoxic rCPE_D48A_ and rCPE_C186A/F91C_ variants were significantly more efficient at altering TEER or FD4 transit than rC-CPE (which lacks the N-terminal domain). Second, these *in vitro* studies detected no consistent differences in TEER changes or FD4 transit effects between rCPE_D48A_ and rCPE_C186A/F91C_, indicating that oligomerization is not important for CPE to cause permeability effects in Caco-2 cells. The overall conclusions from these *in vitro* studies are that, for CPE to cause permeability changes in Caco-2 cell monolayers, i) receptor binding is important, ii) the presence of the N-terminal domain contributes beyond its ability to form a prepore or pore, and iii) cytotoxicity enhances both permeability efficacy and scope.

Some, but not all, of the conclusions regarding CPE permeability effects obtained from the current *in vitro* studies were recapitulated with regard to CPE absorption from the intestines. For example, receptor binding was also found to contribute to efficient intestinal absorption of CPE. Similarly, cytotoxic activity also increased the efficacy of CPE intestinal absorption. Last, binding-capable, noncytotoxic rCPE variants not only affected *in vitro* permeability but also became well-absorbed from the intestines, although longer treatment times were needed to achieve equality with the absorption of cytotoxic rCPE species.

At least two differences were also observed between the *in vivo* vs *in vitro* results. The first difference concerns the effects of rCPE_D48A_ versus rC-CPE on small molecule permeability. Although rCPE_D48A_ caused significantly more effects than CPE_Y306A/315A_ on FD4 permeability *in vitro*, no significant difference was detected between the absorption of EB (*M*_r_ 961) induced by these two rCPE variants *in vivo* (further discussion later). A second difference identified involves the importance, beyond its role in pore formation, of the N-terminal domain for causing permeability effects i.e., whereas rC-CPE caused less *in vitro* effects on FD4 permeability than rCPE_D48A_, the intestinal absorption of rC-CPE and rCPE_D48A_ were similar. Those differences regarding the contributions of N-terminal CPE sequences to noncytotoxic rCPE variant permeability effects could have at least two explanations. The cell culture model is more sensitive than the *in vivo* model, so a modest contribution of N-terminal sequences to *in vivo* intestinal absorption by binding-capable, but noncytotoxic, rCPE variants might not be discernible in the *in vivo* assays, Alternatively, the small intestine is a much more complex environment than a Caco-2 cell monolayer, that is, the small intestine contains multiple cell types, along with factors such as intestinal mucus, that might influence contributions (beyond pore formation and cytotoxicity) of the N-terminal CPE domain to absorption or permeability.

The current study also provides new insights regarding CPE-induced lethal enterotoxemia. First, this work determined that only cytotoxic rCPE species cause lethal enterotoxemia. Because CPE cytotoxicity results from pore formation, this determination is consistent with previous observations that i) in the mouse small intestinal loop model, CPE eventually causes lethal hyperpotassemia (elevated potassium levels in blood) and ii) via pore formation, CPE causes rapid release of potassium analogs from sensitive cultured cells (5, 25, 39).

In addition, a previous study (25) indicated that CPE absorption is important for CPE-induced enterotoxemic lethality. For example, a seroprotection experiment showed that i.v. administration of a neutralizing anti-CPE monoclonal antibody protected mice from CPE-induced enterotoxemic lethality even though this antibody had no effect on the development of intestinal histologic lesions (25). This result and others in that prior study are consistent with the current results showing that the development of histologic lesions is not necessary for CPE absorption, although the presence of those lesions did apparently enhance the efficacy of CPE absorption in the current study. Specifically, rCPE was absorbed more quickly than binding-capable, but noncytotoxic, rCPE variants; however, by 4 h, those noncytotoxic, binding-capable variants became equally well-absorbed as rCPE, even though they still did not cause histologic damage. It is notable that this slow absorption of the noncytotoxic, binding capable variants was not due to a general nonspecific increase in intestinal permeability because these variants did not change EB uptake between 2 and 4 h. The pathological relevance, if any, of demonstrating that receptor binding alone can trigger intestinal CPE absorption is unclear, but one possibility (requiring further study) might be that this binding could begin initiating toxin absorption even prior to the development of CPE-induced histologic damage in the intestines.

There is translational interest in using rC-CPE to, for example, increase drug absorption across epithelia (31). An obvious advantage to using rC-CPE rather than CPE or rCPE for these applied purposes is its lack of cytotoxic properties that could cause side effects *in vivo*. However, the current study determined that cytotoxicity-induced damage facilitates the efficacy of CPE effects on small molecule absorption, i.e., rC-CPE is much less efficient than rCPE at inducing FD4 permeability changes *in vitro* (FD4 transit) or EB absorption *in vivo*. A possible alternative to using rC-CPE for inducing small molecule permeability changes would be to use a full-length, noncytotoxic rCPE variant like rCPE_D48A_. However, although rCPE_D48A_ did work better than rC-CPE for enhancing FD4 transit across Caco-2 cell monolayers, it did not significantly improve EB uptake in the intestines. Despite that observation, the effects of rCPE_D48A_ on small drug permeability *in vivo* might warrant further study in other settings (e.g, the nasoepithelium) or with small molecules other than EB.

Although further studies are needed to fully understand how CPE induces permeability changes, including CPE absorption, the current study provide some initial mechanistic insights. The current results clearly established that, both *in vitro* and *in vivo*, CPE cytotoxicity enhances permeability alterations, particularly for larger molecules like FD40 or CPE. *In vitro*, this permeability enhancement likely involves CPE-induced pore formation causing cellular morphologic damage, which initially involves cell rounding (with likely barrier disruption) and can later progress to include cell detachment that disrupts the monolayer (Fig. 3) (40, 41). It should be noted that, in the current study, rCPE began to induce significant FD4 or TEER permeability effects prior to causing detectable cell detachment from Caco-2 cell monolayers. However, the onset of rCPE-induced FD40 permeability changes overlapped with the development of monolayer damage, suggesting that loss of monolayer integrity may be responsible for CPE alterations in monolayer permeability to large molecules like FD40. The situation is likely more complex in the intestines, where previous studies strongly suggest that histologic damage due to direct CPE-induced cytotoxicity is largely confined to villus tips, with this initial damage then triggering a bystander killing effect to cause histologic damage to the remainder of the villus (42). Together those effects likely disrupt the integrity of the CPE-treated epithelial barrier *in vivo* to increase permeability.

How noncytotoxic, but binding-capable, rCPE variants can induce CPE absorption and alter *in vitro* FD4 permeability without causing cell death or cell detachment leading to monolayer disruption *in vitro* or intestinal histologic damage is less clear. The more subtle effects of these noncytotoxic, binding capable variants could conceivably involve the reported ability of rC-CPE to disrupt tight junctions (TJs) (43). For example, rC-CPE was shown to disrupt TJs of MDCK cells, although this only occurred when applied to the basolateral surface (43). Whether rC-CPE can cause similar TJ damage when applied to the apical surface of Caco-2 cells or the intestines awaits further study. It would also be unclear how TJ damage would enhance intestinal CPE absorption without causing an increase in EB uptake. Given the interest in using noncytotoxic, binding-capable rCPE variants for drug delivery future studies should further investigate the mechanisms by which those variants alter permeability, including inducing their own absorption from the intestines.

## MATERIALS AND METHODS

### Cell culture.

Caco-2 human colorectal adenocarcinoma cells (Caco-2) were maintained in Eagle’s minimum essential medium (Lonza, Walkersville, MD, USA) supplemented with 10% fetal bovine serum (Gibco, Life Technologies Corporation (Grand Island, NY, USA, 1% MEM nonessential amino acids (HyClone, GE Healthcare Life Sciences, Logan, UT, USA), 100 μg mL^−1^ penicillin-streptomycin (Corning, Mediatech Inc, Manassas, VA) and 1% glutamine (Life Technologies Corporation, Grand Island, NY). These cells were routinely maintained at 37°C in an atmosphere containing 5% CO_2_. For experiments, Caco-2 cells were inoculated into Transwell Permeable Supports, as described below.

### Construction and purification of recombinant CPE (rCPE) species.

This study used several different rCPE species, which are listed in Table 1. To produce these species, open reading frames (ORFs) encoding rCPE or the rCPE variants rCPE_D48A_, rCPE_C186A/F91C_, rCPE_C186A_, rC-CPE (amino acids 184–319 of native CPE) and rCPE_Y306A/L315A_ were synthesized by GenScript, and their identity was then confirmed at GenScript by sequencing using the primers shown in Table 2. Each ORF was then individually ligated into the expression plasmid pET-45(b+) between the SacI and AvrII sites. Each resultant plasmid was separately transformed into E. coli HMS 147 (purchased from New England Biolabs).

**TABLE 2:** Primers used for sequencing of rCPE species

CPE species	Primers
rCPE	T7: TAATACGACTCACTATAGGG
	T7ter: TGCTAGTTATTGCTCAGCGG
rCPE_D48A_	T7: TAATACGACTCACTATAGGG
	T7ter: TGCTAGTTATTGCTCAGCGG
rCPE_C186A_	T7: TAATACGACTCACTATAGGG
	T7ter: TGCTAGTTATTGCTCAGCGG
rCPE_C186A/F91C_	T7: TAATACGACTCACTATAGGG
	T7ter: TGCTAGTTATTGCTCAGCGG
rC-CPE	T7: TAATACGACTCACTATAGGG
	T7ter: TGCTAGTTATTGCTCAGCGG
rCPE_Y306A/L315A_	T7: TAATACGACTCACTATAGGG
	T7ter: TGCTAGTTATTGCTCAGCGG

**FIG 10:**
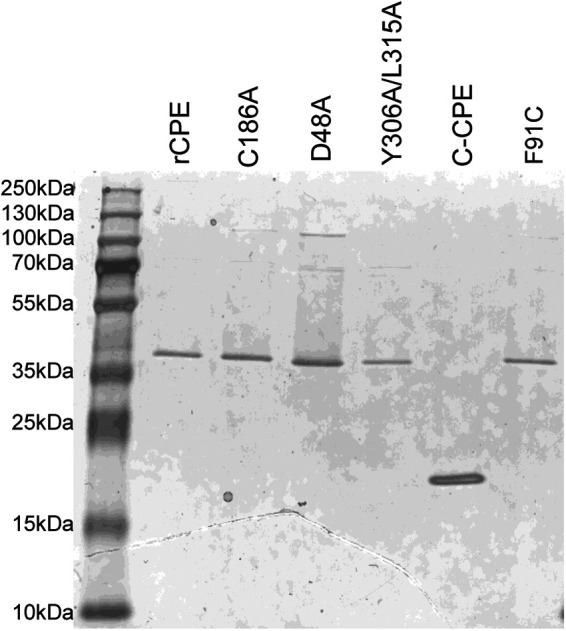
Evaluation of rCPE species purity. After purification by metal affinity purification (see Materials and Methods), ~5 μg of each purified rCPE species was electrophoresed on a 12% acrylamide gel containing SDS and then stained with G250 Coomassie blue (Bio Rad Laboratories).

Each His_6_-tagged rCPE species was then purified from lysates of recombinant E. coli transformants. For this purpose, 1 L of Luria Bertani (LB, Fisher Bioreagent) broth was inoculated with 8 mL of an overnight LB broth culture of E. coli expressing the desired rCPE species. After shaking the cultures for 2 h at 37°C, expression of each rCPE species was induced by the addition of isopropyl-β-d-thiogalactopyranoside to a final concentration of 1 mM. After the induced culture was incubated for 3.5 h at 37°C with shaking, the bacteria were harvested by centrifugation. Pellets were resuspended in lysis buffer (50 mM NaH_2_PO_4_, 300 mM NaCl, 10 mM Imidazole, pH 8.0). The bacterial suspension was then treated with 1 mg/mL of lysozyme for 1 h and sonicated by 3 cycles of 10 sec pulse with 30 sec off using a Sonicator 3000 (Misonix, Farmingdale, NJ). Cell debris were then pelleted by centrifugation and each supernatant was collected and incubated with equilibrated Nickel resin (Qiagen) for 1 h at 4°C, with rotation. The resin was then washed twice with wash buffer (50 mM NaH_2_PO_4_, 300 mM NaCl, 20 mM Imidazole, pH 8.0) and each rCPE species was eluted from the resin with elution buffer (50 mM NaH_2_PO_4_, 300 mM NaCl, 250 mM Imidazole, pH 8.0). Protein-containing fractions from each elution were pooled and desalted using a Zeba Spin desalting column 7K MWCO (Thermo Scientific). The harvested rCPE species from each purification was quantified using a Bicinchonic Acid (BCA) protein kit assay (Thermo Scientific, Rockford, IL). The presence of the rCPE species in each preparation was confirmed by CPE Western blotting. All rCPE species were purified to near homogeneity (Fig. 10), as assessed by SDS-PAGE and Coomassie blue staining (5).

### Cytotoxicity measurement.

Caco-2 cells in Transwell Permeable Supports (12mm^2^ insert, 12-well plate, 0.4 μm polyester membrane, Costar) were cultured to confluence for 14 days, with a change of medium every 2 days. After two washes with warm HBSS buffer, the top chamber of each Transwell was treated with 0.5 mL of warm HBSS containing the specified concentrations of each rCPE species. One mL of fresh HBSS buffer (no rCPE species) was also added to the bottom chamber of each Transwell plate. After incubation of these cultures at 37°C for specified time points, the supernatants were collected, and cytotoxicity was assessed using an LDH cytotoxicity detection kit (Roche) according to the manufacturer’s instructions. HBSS with or without 1% Triton X-100 (Sigma) were used as negative and positive controls, respectively.

### Cell microscopy.

Each Confluent Caco-2 cell monolayer treated with rCPE species, as described above for the cytotoxicity experiments, was also visually monitored for the development of monolayer damage. In addition, microscopic photographs were taken to document the development of monolayer damage in a representative well for each treatment over time (Fig. 4 A–D) or after overnight treatment (Fig. 4E). Images were obtained using a Nikon Eclipse Ti inverted microscope with Meta Morph (64 bit) Version 7.7.4.0 software (Molecular Devices Inc, San Jose CA). All photomicrographs were taken using ×100 magnification.

### Measurement of rCPE species effects on transepithelial electrical resistance (TEER) and fluorescently labeled dextran permeability in Caco-2 cells.

Caco-2 cells were seeded at a density of 10^4^ cells/well in Transwell permeable supports and grown, as described above, for 2 weeks. The Transwells were then challenged with specified concentrations of each rCPE species for the indicated times. Specified higher doses of rC-CPE or rCPE_Y306A/L315A_ were also used to measure the TEER on Caco-2 cells. TEER was then measured using the Millicell ERS-2 (Electrical Resistance System) (Sigma) and resistance was calculated using the following equation (38):

$$\text{Unit Area Resistance}=\text{Resistance }\left(Ω\right)\text{ × Effective Membrane Area }\left(\text{c}{\text{m}}^{2}\right)$$

To measure the permeability effects of rCPE species, the medium was gently removed, and cells were washed twice with HBSS buffer added to both the upper and lower chambers. The washed inserts were then transferred to fresh 12-well plates and 0.5 mL of medium containing a specified concentration of each rCPE species and a FITC-labeled fluorescent dextran (FD), either FD4 (Sigma Chemical, final concentration of 1 mg/mL) or FD40 (Sigma Chemical, at a final concentration of 1 mg/mL) were added into the upper chamber of each Transwell. In addition, 1.5 mL of HBSS were added to the lower chamber of each well. After a 1 h of incubation at 37°C, the transferred FD concentration in the lower chamber was determined using a BioTek Synergy Flurorescence multi plate reader (BioTek, Winooski, VT), with excitation and emission of 485 nm and 530 nm, respectively.

Fluorescent measurement of FD permeabilty as calculated based on the below given formula (38):

$$\text{Fluorescence }=\text{ Abs }\left(1/\text{Area of Transwell}\right)\text{ × }\left(\text{sample}/3,\text{600}\right)$$

### Comparison of fluorescently labeled rCPE, rCPE_Y306A/L315A_, and rCPE_D48A_ binding to Caco-2 cells.

As described previously for rCPE (38), rCPE, rCPE_Y306A/L315A_, and rCPE_D48A_ were covalently labeled with Alex-Fluor 488 (AF488) according to the instructions provided in the Invitrogen Alexa-Fluor 488 Protein Labeling Kit. The cytotoxic properties of each AF488-labeled rCPE species was then assessed using the LDH cytotoxicity detection kit (Roche).

To compare the binding properties of these AF488-labeled rCPE species, 2-week-old Transwell cultures of Caco-2 cells were treated for 1 h at 37°C with 5 μg/mL of an AF488-labeled rCPE species. The cells were washed twice with HBSS buffer and then lysed with RIPA buffer (5 mM TrisHCl pH 7.6, 150 mM NaCl, 1% NP-40, 1% sodium deoxycholate, and 0.1% SDS). Lysates were collected to measure fluorescence using a multiplate reader (BioTek, Winooski, VT), with excitation and emission of 485 nm and 530 nm, respectively.

### Western blot analysis of CH-1 CPE complex formation.

Confluent Caco-2 cells were treated at 37°C with HBSS containing 1 μg/mL of rCPE or rCPE_Y306A/L315A_ for 60 min, scraped, washed once, and lysed with RIPA buffer with Benzonase (EMD, Millipore Corp.). Lysates were electrophoresed on SDS-containing gels with 6% polyacrylamide and then transferred onto a nitrocellulose membrane (Bio-Rad). To detect the presence of SDS-resistant large complex, Western immunoblotting was performed on this membrane, as described previously (4).

### IACUC.

All experiments involving mice were reviewed and approved by the University of California Davis Institutional Animal Care and Use Committee (protocol 21554).

### Effects of rCPE species on general intestinal permeability during a 2- or 4-h incubation period.

To compare effects of the rCPE species on general intestinal permeability, a previously described approach (44) based on EB quantification in the intestine of rats was used, with modifications. Segments of ~10 cm of mouse small intestine were surgically ligated in mice under general anesthesia as previously described (25, 45). These intestinal loops were performed in five groups of mice (*n* = 10 per group) and challenged with 1 mL of HBSS containing 3% EB combined with a 2.85 μM concentration of rCPE, rC-CPE, rCPE_D48A_, or rCPE_Y306A/315A_. The fifth group received EB only (no rCPE variants). After 2 h or 4 h of incubation, the mice were euthanized. Samples of the intestinal loops were collected and washed to remove luminal contents with saline solution. The intestinal loops were then washed again three times with 6 mM L-acetylcysteine to remove any excess mucus. The intestinal tissue was dried on filter paper for 24 h at 4°C and then incubated with 1 mL of formamide at 50°C for 24 h. The eluted dye was quantified at wavelength 620 nm in the formamide solution.

### Effects of rCPE species on CPE absorption and intestinal damage.

From mice used in the same experiment described in the preceding section, samples of blood were collected from the heart under general anesthesia or immediately before euthanasia. The presence of rCPE variants in serum from those samples was quantified using a commercial ELISA kit (Techlab), as previously described (45). This involved comparing the ELISA value for each rCPE variant present in a serum sample against an ELISA standard curve constructed using the same variant. In addition, samples of challenged intestinal loop were also collected and fixed in buffered formalin (10%), pH 7.2, for 72 h and then processed to produce 4-μm sections stained with hematoxylin and eosin (H&E). Histologic samples were examined in a blind fashion by a veterinary pathologist. Intestinal damage was assessed and an ordinal scale from 0 (no lesions) to 4 (severe lesions) was assigned to each section analyzed. Microscopic changes considered for this score included: blunting of villi, desquamation of enterocytes, death of enterocytes, presence of inflammatory cells, dilatation of lymphatic vessels and edema of the submucosa.

### Enterotoxemia assay.

Ligated intestinal loops were created in mice to evaluate the systemic effects of rCPE species. Four groups of mice (*n* = 10 per group) received direct inoculations into their intestinal loop, as described in the previous section, with 1 mL of HBSS containing a 2.85 μM concentration of rCPE, rC-CPE, rCPE _D48A_, rCPE_Y306A/315A_, or buffer only. The mice were incubated for up to 4 h with these mice being anesthetized throughout the experiment. Death and survival were recorded during this period. Mice that did not die by the end of the 4 h of incubation were humanely euthanized.

### Statistical analyses.

In the *in vitro* studies, all experiments were independently repeated three times and values are shown as mean ± standard deviation. For statistical analysis, one-way analysis of variance (ANOVA) was performed using GraphPad Prism, version 6 (GraphPad, San Diego, CA). Differences were considered significant when the *P* value was less than 0.05.

All statistical analyses for the *in vivo* experiments were performed using R (v3.3.1). For comparison of treatment, one-way ANOVA was applied with *post hoc* analysis using Tukey’s multiple-comparison test for three or above treatment. Student *t*-test analysis was used for two group numbers comparison. Histopathological scores were compared by using the nonparametric Kruskal-Wallis test, followed by the Dunn test as *post hoc* analysis. Differences were considered significant when the *P* value was less than 0.05.
